# Supplementary material for: Chromothripsis during telomere crisis is independent of NHEJ, and consistent with a replicative origin
Source: Genome Res. 2019 May;29(5):737–49. doi: 10.1101/gr.240705.118 (PMC6499312; doi:10.1101/gr.240705.118)
Supplement: Supplemental Material [file supp_gr.240705.118_Supplemental_file_1.zip › contigs/annotated_contigs/DB55/contig.2.DB55_length_366_mean_cov_3.10655737705.docx]

**DB55_length_366_mean_cov_3.10655737705**

```
GTCTCAAACTTCTGACCTCTGGTGCTCCGCCCGCTTCGGCCTCCTAAAGTTCTGGGATTACAGGTGTTAACCACCATA|CCTG|GCTAT
 >chr6:163886876-163886958 - E=7e-26                                           >chr7:14
ATTCATTTTTATATGTATATAAAAATATATGTATATATATGTGTATATATGTATATATATGTGTATGTGTGTTTGTGTGTGTGTAATAT
4830876-144831164 + E=3e-162
ATAAACATATGCTCACAGCAATACCTGCAATCTCAACTCAACACCATAGGATTTATTTGAGTTTTTTCTCTTTTCATTTATGTATATAC

TGCTGATATGGTTTGGCTGTGTCCGCAGCCAAATCTCATCTTGAATTGTAGCTCCCATAATTCCTACGTGTTGTGGGAGGGACCTGGTA

GGAGATAATTGA
```
